# Deep immunophenotyping reveals that autoimmune and autoinflammatory disorders are spread along two immunological axes capturing disease inflammation levels and types

**DOI:** 10.1136/ard-2023-225179

**Published:** 2024-01-05

**Authors:** Nicolas Tchitchek, Marie Binvignat, Alexandra Roux, Fabien Pitoiset, Johanna Dubois, Gwendolyn Marguerit, David Saadoun, Patrice Cacoub, Jérémie Sellam, Francis Berenbaum, Agnès Hartemann, Chloé Amouyal, Roberta Lorenzon, Encarnita Mariotti-Ferrandiz, Michelle Rosenzwajg, David Klatzmann

**Affiliations:** 1 INSERM UMRS 959, Immunology-Immunopathology-Immunotherapy (i3), Sorbonne Université, Paris, France; 2 Clinical Investigation Center for Biotherapies (CIC-BTi) and Immunology-Inflammation-Infectiology and Dermatology Department (3iD), Assistance Publique–Hôpitaux de Paris, Hôpital Pitié-Salpêtrière–Charles Foix Hospital, Paris, France; 3 INSERM U938, Rheumatology Department, Saint-Antoine Hospital, AP–HP, Sorbonne Université, Paris, France; 4 Department of Internal Medicine and Clinical Immunology and Immunology-Inflammation-Infectiology and Dermatology Department (3iD), Reference Center for Autoinflammatory Disorders (CEREMAIA); Reference Center for Systemic Autoimmune Diseases, Paris, France; 5 Diabetology-Metabolism Department, AP–HP, Institut Hospitalo-Universitaire de Cardiometabolisme et Nutrition (ICAN), Pitié-Salpêtrière-Charles Foix Hospital, Sorbonne Université, Paris, France; 6 Institut Universitaire de France (IUF), Paris, France

**Keywords:** Immune System Diseases, Autoimmunity, Inflammation, T-Lymphocyte subsets

## Abstract

**Objectives:**

Based on genetic associations, McGonagle and McDermott suggested a classification of autoimmune and autoinflammatory diseases as a continuum ranging from purely autoimmune to purely autoinflammatory diseases and comprising diseases with both components. We used deep immunophenotyping to identify immune cell populations and molecular targets characterising this continuum.

**Methods:**

We collected blood from 443 patients with one of 15 autoimmune or autoinflammatory diseases and 71 healthy volunteers. Deep phenotyping was performed using 13 flow cytometry panels characterising over 600 innate and adaptive cell populations. Unsupervised and supervised analyses were conducted to identify disease clusters with their common and specific cell parameters.

**Results:**

Unsupervised clustering categorised these diseases into five clusters. Principal component analysis deconvoluted this clustering into two immunological axes. The first axis was driven by the ratio of LAG3+ to ICOS+ in regulatory T lymphocytes (Tregs), and segregated diseases based on their inflammation levels. The second axis was driven by activated Tregs and type 3 innate lymphoid cells (ILC3s), and segregated diseases based on their types of affected tissues. We identified a signature of 23 cell populations that accurately characterised the five disease clusters.

**Conclusions:**

We have refined the monodimensional continuum of autoimmune and autoinflammatory diseases as a continuum characterised by both disease inflammation levels and targeted tissues. Such classification should be helpful for defining therapies. Our results call for further investigations into the role of the LAG3+/ICOS+ balance in Tregs and the contribution of ILC3s in autoimmune and autoinflammatory diseases.

**Trial registration number:**

NCT02466217.

**WHAT IS ALREADY KNOWN ON THIS TOPIC**

- Autoimmune and autoinflammatory diseases represent a heterogeneous group of disorders whose nosology is unclear, and for which there are no curative treatments.
- It has been proposed that these disorders are spread along a continuum ranging from purely autoimmune to purely autoinflammatory diseases.
- We evaluated this hypothesis by conducting deep immunophenotyping of blood cells from patients with one of 15 autoimmune and autoinflammatory diseases*.*

**WHAT THIS STUDY ADDS**

- Using unsupervised clustering, we found that diseases were categorised into five clusters.
- Using principal component analysis, we identified two immunological axes associated with disease inflammation and disease type/localisation rather than a monodimensional continuum of diseases.
- The ratio of LAG3+ and ICOS+ in regulatory T lymphocytes (Tregs) was associated with disease inflammation levels.
- Activated Treg subsets and type 3 innate lymphoid cells (ILC3s) were associated with the types/localisations of diseases.

**HOW THIS STUDY MIGHT AFFECT RESEARCH, PRACTICE OR POLICY**

- We reveal a novel nosology of autoimmune and autoinflammatory diseases based on deep immunophenotyping.
- These results call for further investigations into the LAG3+/ICOS+ balance in Tregs as well as activated Tregs and ILC3s in autoimmune and autoinflammatory diseases.
- This raises the question of whether patients from the same identified clusters may benefit from the same therapies.

## Introduction

Autoimmune diseases (AD) and autoinflammatory diseases (AIF) are pathological conditions arising from the imbalance between immune tolerance and activation.^1^ AD and AIF represent more than 80 different heterogeneous disorders affecting up to 8% of the world’s population.^2^ Despite the prevalence and heterogeneity of these diseases, the pathophysiology and the nosology of these diseases remain largely elusive, and despite numerous treatment option we currently lack curative ones.^3^

AD and AIF can be classified according to several criteria,^4^ and are mostly classified according to a combination of clinical and biological feature sets. A generally accepted notion is that AD predominantly stems from dysregulations of adaptive immunity and that AIF arises primarily from dysregulations of innate immunity. However, it has been proposed that AD and AIF could rather lie on a continuum ranging from autoimmune to autoinflammatory, with different contributions of both the innate and the adaptive immune responses.^5–8^ Additionally, these disorders can be classified based on their types/localisations (such as joint, blood vessel, bowel, metabolism, or muscle), which suggest a contribution of tissue-specific factors in the proposed continuum.^4 7 9^

Only few studies have evaluated the differences between AD and AIF in a systematic manner, limiting the validation of the continuum hypothesis and the identification of immunological components that could be responsible for their similarities or differences. To revisit the nosology of AD and AIF, we initiated the Transimmunom observational clinical trial involving 443 patients with one or more of 15 disorders ranging from pure AD to pure AIF.^10^ Patients’ medical history and status were recorded, and deep phenotyping performed. We report here the results of the deep cytometry immunophenotyping that analysed more than 600 innate and adaptive immune cell parameters (both absolute cell counts and percentages) from patient’s blood. Unsupervised and supervised analyses were conducted with the purpose of: (1) evaluating the hypothesis of an AD to AIF continuum, (2) identifying clusters of diseases along with their shared and specific cell parameters and (3) characterising potential biomarkers.

## Materials and methods

### Study design and participants

**Figure 1:**
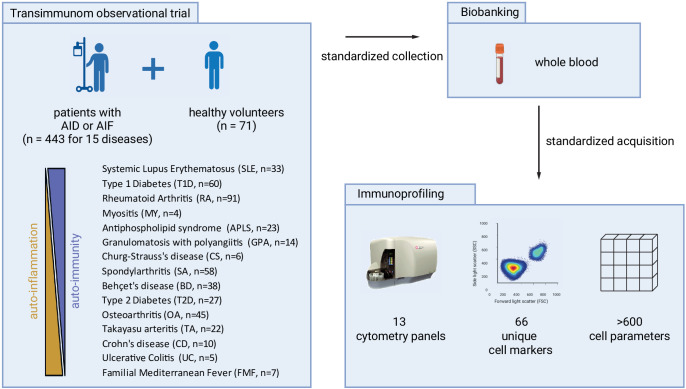
Experimental design of the Transimmunom observational clinical trial. We standardly collected whole blood from 447 patients having 15 autoimmune or autoinflammatory diseases enrolled in the Transimmunom observational clinical trial, and from 71 healthy volunteers. Disorders ranged from pure autoinflammatory to pure autoimmune diseases. The abbreviation and the number of patients associated with each disease are indicated. After quality control, samples were profiled using 13 flow cytometry panels quantifying a total of 66 unique cell markers to characterise more than 600 cell parameters (ie, absolute cell counts and percentages of cell populations). AID, autoimmune disease; AIF, autoinflammatory disease.

Building on on the Transimmunom clinical trial,^10^ we collected peripheral blood from 443 patients (enrolled from 2015 to 2022) who were affected by 15 distinct ADs or AIFs or related conditions, as well as blood from 71 healthy volunteers (HV) to serve as a reference condition (figure 1). Our screened disorders ranged from pure AIF to pure AD and included different types of disorder activities without any threshold of disease activity. These disorders included arthritis disorders as Behçet’s disease (BD; n=38), knee osteoarthritis (OA; n=45), rheumatoid arthritis (RA; n=91), spondyloarthritis (SA; n=58) and systemic lupus erythematosus (SLE; n=33); blood vessel disorders as antiphospholipid syndrome (APLS; n=23), Churg-Strauss disease (CS; n=6), granulomatosis with polyangiitis (GPA; n=14) and Takayasu arteritis (TA; n=22); metabolic disorders as type 1 diabetes (T1D; n=60) and type 2 diabetes (T2D; n=27); muscle disorder as myositis (MY; n=4); inflammatory bowel diseases (IBD) as Crohn’s disease (CD; n=10) and ulcerative colitis (UC; n=5); and IBD-like diseases as familial Mediterranean fever (FMF; n=7). OA and T2D were primarily included in this disease spectrum as ‘benchmarks’ for RA and T1D, but also considering the recent observations that suggest that these diseases have autoimmune and autoinflammatory components.^11–15^ Criteria used for disease diagnostics are indicated in online supplemental table S1. Main treatments associated with diseases are indicated in online supplemental table S2. These treatments included classical therapies provided to patients with AD and AIF, such as insulin for patients with T1D, oral antidiabetics for patients with T2D, hydroxychloroquine for patients with SLE, non-steroidal anti-inflammatory drugs (NSAIDs) for patients with AS, glucocorticosteroids for patients with BD, CS, GPA and TA, along with synthetic disease-modifying antirheumatic drugs, biological disease-modifying antirheumatic drugs and NSAIDs.

10.1136/ard-2023-225179.supp1 Supplementary data

### Deep immunophenotyping

Cytometry profiling of patient blood samples was performed using 12 flow cytometry panels of 10 cell markers each, as previously described.^16^ An additional cytometry panel targeting innate lymphoid cells (ILC) was used. In detail, these panels were designed to perform advanced analysis of T cells, B cells, natural killer (NK) cells, mucosal-associated invariant T (MAIT) cells, myeloid cells, monocytes and dendritic cells (DC). A set of three panels was specifically created to analyse the activation, migration and memory phenotype of T cells. One panel was specifically designed for investigating CD4+ T cell polarisation, whereas two panels primarily concentrated on studying the phenotype of regulatory T cells. Other panels were designed to explore B cells, NK cells, monocytes, dendritic cells, MAIT cells and myeloid-derived suppressor cells. We also developed an extra panel to identify the main immune cell populations. This panel included numeration beads, allowing to determine the absolute counts of all populations, while serving as a reference tube that allows the calculations of absolute counts in all other panels by extrapolation from shared populations. All acquisitions were performed on a Gallios cytometer (Beckman Coulter) maintained daily according to the manufacturer’s recommendations with Flow Check Pro and Flow Set Pro fluorospheres. Cell parameters were defined as previously described,^16^ and were obtained using manual gating using Kaluza V.1.3 software (Beckman Coulter). Regulatory T lymphocytes (Tregs) were defined as CD25+/CD127− cells among T cells. The expression of FoxP3 was also measured in one cytometry panel. ILCs were defined as CD127+ cells and based on a negative linage comprising CD4, CD3, CD14, CD19, CD34, TCRγδ, CD1a, TCRαβ, CD11c, CD94, CD123, FcεR1a and CD303. ILC1s were defined as CD294−/CD117−, ILC2s were defined as CD294+ and ILC3s were defined as CD294−/CD117+. More than 600 cell parameters were quantified using such deep immunophenotyping of innate and adaptive cell populations.

As previously described, Duraclone technology was used to standardise the staining procedures, which provides the possibility to use custom-designed panels of antibodies that are dried and precoated in individual tubes for direct labelling of blood. Moreover, we evaluated the coefficient of variability of measurements of populations that are shared by different panels. For validation of the regulatory T cell measurements, we assessed the correlation between the values obtained with or without the FoxP3 marker.^16^

### Univariate analyses

Due to the unbalanced nature of the number of patients per biological condition, both Cliff’s Delta effect size and two-way non-parametric Wilcoxon test were used to identify cell parameters that were significantly differentially abundant between conditions. Cliff’s Delta is a non-parametric measure of effect size that is used in statistics to quantify the magnitude of the difference between two groups or conditions.^17^ Cliff’s Delta ranges from −1 to +1, where values closer to −1 indicate a large effect size in favour of the first group, values closer to +1 indicate a large effect size in favour of the second group and values close to 0 indicate a small or negligible effect size. Cell parameters with an absolute value of Cliff’s Delta effect size higher than 0.33—corresponding to a medium magnitude change—and with a p value lower than 0.05 were considered statistically significant.

### Multivariate analyses

Hierarchical agglomerative clustering and dendrogram representations represented with heatmaps were constructed based on the Euclidean distances and using the Ward’s linkage method. Principal component analyses (PCA) were generated using the FactoMineR R package using unscaled Cliff’s Delta effect size values of cell parameters in each disease relative to HV. Cell parameters with an eigenvalue lower threshold than −0.5 or higher than 0.5 in one axis were considered to be associated with PCA axes. The identification of disease clusters along PCA axes was performed using k-means clustering. The optimal number of clusters was determined using the NbClust R package. Multidimensional scaling (MDS) representation was generated using the MASS R package based on Cliff’s Delta effect size values of cell parameters in each disease relative to HV. The coexpression network was constructed using the Spearman coefficient of correlation using an absolute threshold of 0.6. Classification decision trees were generated using the partykit R package based on all available cell parameters, using a maximal depth parameter of 5 and a minimal bucket size parameter of 10.

### Patient and public involvement

Patients or the public were not involved in the design, conduct, reporting or dissemination plans of our research.

### Data availability

The relative percentage or absolute count of the 224 cell parameters differentially abundant in at least one disease relative to HV is available on the Zenodo open repository through DOI: 10.5281/zenodo.10364382.

## Results

### Experimental design and patient demographic characteristics

We included in this study all the 443 patients and 71 HVs from the Transimmunom trial from which blood samples were available (figure 1). The list of diseases included in this study and patient demographics are presented in table 1. Diseases ranged from purely AD to purely AIF. We also included diseases such as OA and T2D that were primarily used as ‘benchmaks’ for RA and T1D, respectively, and for which accumulating observations suggest autoimmune or autoinflammatory components in their aetiologies.^11–15^ Across all diseases, the mean age was 44.42±15.30 years, the mean body mass index (BMI) was 25.82±9.68 and the sex ratio was 57.70% towards females. There were no differences in age, BMI or sex ratio of patients relative to HV, except when expected (female bias for RA, SLE and TA; high BMI for APLS, OA, TA, T2D and UC; age bias for APLS, CS, GPA, MY, TA, OA, RA and T2D). The average time from diagnosis was 5.11±6.91 years (table 1). More than 600 innate and adaptive immune cell populations from patient’s blood were characterised using multiple flow cytometry panels that were designed to perform advanced analysis of T cells, B cells, NK cells, MAIT cells, myeloid cells, monocytes, dendritic cells and ILCs.^16^

**Table 1:** Characteristics of groups of individuals included in the study

Condition	Abbreviation	Patients (n)	Age mean±SD (P value)	BMI mean±SD (P value)	Sex F/M (P value)	Years since disease onset mean±SD
Healthy volunteers	HV	71	37.23±12.18	23.90±3.29	36/35	
Antiphospholipid syndrome	APLS	23	57.23±12.42 (<0.0001)	26.66±4.75 (<0.0150)	9/13 (NS)	4.67+4.10
Behçet’s disease	BD	38	36.08±11.64 (NS)	23.89±3.97 (NS)	12/25 (NS)	2.84+2.91
Crohn’s disease	CD	10	27.70±7.65 (NS)	22.51±3.31 (NS)	4/6 (NS)	3.60+2.59
Churg-Strauss disease	CS	6	61.00±8.94 (0.0140)	22.46±5.57 (NS)	5/1 (NS)	5.17+4.17
Familial Mediterranean fever	FMF	7	36.43±16.15 (NS)	24.93±3.03 (NS)	4/3 (NS)	24.83+8.33
Granulomatosis with polyangiitis	GPA	14	49.86±14.78 (0.0045)	26.39±5.64 (NS)	8/6 (NS)	2.71+2.61
Myositis	MY	4	58.00±1.83 (<0.0023)	23.52±3.76 (NS)	3/1 (NS)	1.00+0.82
Osteoarthritis	OA	45	64.60±9.68 (<0.0001)	29.38±7.11 (<0.0001)	28/15 (NS)	7.53+7.77
Rheumatoid arthritis	RA	91	49.11±13.43 (<0.0001)	27.78±20.31 (NS)	72/17 (<0.0001)	4.95+7.14
Spondyloarthritis	SA	58	39.54±12.03 (NS)	24.95±4.49 (NS)	25/32 (NS)	5.09+7.63
Systemic lupus erythematosus	SLE	33	40.81±10.50 (NS)	23.36±4.05 (NS)	29/3 (<0.0001)	5.72+5.98
Type 1 diabetes	T1D	60	34.25±11.26 (NS)	23.76±3.18 (NS)	26/34 (NS)	2.70+2.36
Type 2 diabetes	T2D	27	51.30±12.93 (<0.0001)	32.41±6.42 (<0.0001)	11/16 (NS)	2.22+2.87
Takayasu arteritis	TA	22	48.05±13.47 (0.0015)	26.95±4.82 (<0.0025)	18/4 (<0.0128)	4.73+3.69
Ulcerative colitis	UC	5	34.80±19.89 (NS)	20.95±2.06 (0.0343)	2/3 (NS)	3.40+1.95

For each group of patients included in the study or for healthy individuals, the abbreviation of the biological condition, the number of corresponding patients, the average age, the average body mass index (BMI), the sex ratio and the average number of years since the disease onset are indicated. Significant differences in age, BMI or sex ratio compared with healthy individuals are indicated with their p values computed using the Student’s t-test or the Fisher’s exact test.

APLS, antiphospholipid syndrome; BD, Behçet’s disease; CD, Crohn’s disease; CS, Churg-Strauss disease; FMF, familial Mediterranean fever; GPA, granulomatosis with polyangiitis; HV, healthy volunteer; MY, myositis; OA, osteoarthritis; RA, rheumatoid arthritis; SA, spondyloarthritis; SLE, systemic lupus erythematosus; TA, Takayasu arteritis; T1D, type 1 diabetes; T2D, type 2 diabetes; UC, ulcerative colitis.

### Identification of five clusters of AD or AIF

We first identified cell parameters (absolute cell counts or percentages) differentially abundant in each disease relative to HV (online supplemental figure S1A). Based on our panels, we observed that the diseases had a stronger impact on adaptive immunity than on innate immunity (online supplemental figure S1B,C).

**Figure 2:**
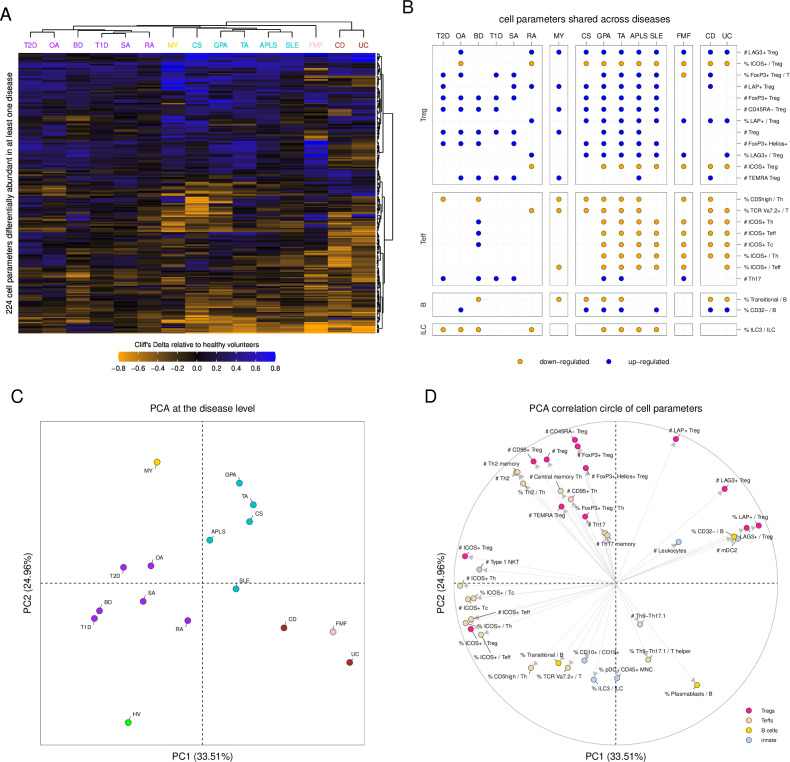
Disorders are gathered into five distinct clusters having each shared and specific immunological contributions. (A) Heatmap representation of Cliff’s Delta effect size measurements of cell parameters statistically different in at least one disease relative to healthy volunteers. Cliff’s Delta is a non-parametric effect size measure that quantifies the difference between two groups, with a range from −1 (all values in one group are lower than those in the other group) to 1 (all values in one group are larger than those in the other group), and 0 indicating no difference between the groups. Effect sizes are represented using a coloured gradient scale ranging from −0.8 to 0.8. Cell parameters downregulated in disease are represented in orange, and cell parameters upregulated in diseases are represented in blue. Unsupervised hierarchical clustering was used to automatically gather diseases and cell populations. Diseases are coloured based on the five disease clusters identified by the hierarchical clustering. (B) Dot plot representation showing the cell parameters found to be significantly impacted in at least seven diseases relative to healthy volunteers. Cell parameters are gathered by main immunological families (ie, Teffs, Tregs, B cells and ILCs). Diseases are ordered and coloured based on the five identified disease clusters. Significantly downregulated cell parameters are indicated in orange, and significantly upregulated cell parameters are indicated in blue. (C) Principal component analysis based on Cliff’s Delta values of each group of patients relative to the healthy condition. The percentages of variance information captured by the two first components are indicated along each axis. Conditions are coloured based on the five disease clusters identified by the hierarchical clustering. (D) Correlation circle showing cell parameters associated with the two first principal components (PC1). Selected parameters are coloured based on their immunological families (ie, Teffs, Tregs, B cells and innate). In such representation, each variable is represented by a dot and an arrow, with its coordinates corresponding to its correlation with PC1 (x-axis) and PC2 (y-axis). The closer the point is to the edge of the circle, the stronger its contribution to the respective principal components. Variables with arrows pointing in the same direction (acute angle between them) have a positive correlation. The closer the angle is to 0 degree, the stronger the correlation is. APLS, antiphospholipid syndrome; BD, Behçet’s disease; CD, Crohn’s disease; CS, Churg-Strauss disease; FMF, familial Mediterranean fever; GPA, granulomatosis with polyangiitis; HV, healthy volunteer; ILC, innate lymphoid cell; MY, myositis; OA, osteoarthritis; PCA, principal component analysis; RA, rheumatoid arthritis; SA, spondyloarthritis; SLE, systemic lupus erythematosus; T1D, type 1 diabetes; T2D, type 2 diabetes; TA, Takayasu arteritis; UC, ulcerative colitis.

Unsupervised hierarchical clustering classified the 15 diseases into five clusters based on the set of 224 cell parameters significantly different relative to HV (figure 2A). The first cluster (C1) included BD, OA, SA, RA, T1D and T2D. The second cluster (C2) consisted solely of MY. The third cluster (C3) encompassed APLS, CS, GPA, SLE and TA. The fourth cluster (C4) contained only FMF. Lastly, the fifth cluster (C5) included CD and UC.

We identified a set of 23 cell populations that were impacted by at least seven diseases relative to HV (figure 2B). These cell populations were mainly associated with Tregs, effector T lymphocytes (Teffs), B cells and ILCs. Remarkably, Treg subsets were consistently upregulated in all diseases relative to HV, except for the percentages of Inducible T-cell Costimulator (ICOS)+ Tregs among Tregs that were downregulated in 10 diseases (APLS, CD, CS, FMF, GPA, OA, RA, SLE, TA, and UC). The absolute counts of ICOS+ Tregs were downregulated in eight diseases (APLS, CD, FMF, GPA, RA, SLE, TA, and UC). Teff subsets were mainly downregulated in almost all diseases relative to HV, except for Th17-associated Teff cell parameters that were upregulated in seven diseases (BD, FMF, GPA, T1D, T2D, TA, and SA). The percentages of CD32− cells and transitional cells among B cells were downregulated in seven diseases (CD, CS, GPA, OA, SLE, TA, and UC). The percentage of ILC3 among ILCs was found to be consistently downregulated in eight diseases (APLS, BD, GPA, OA, RA, SLE, T2D, and TA).

To deconvolute the identified disease clustering, and to understand its driving components, we used PCA. This unsupervised analysis captured around 34% of the variance information in its first component (PC1) and around 25% of the variance information in its second component (PC2). The PCA confirmed the hierarchical clustering (figure 2C). A set of 39 cell parameters, which mainly involved Teffs, Tregs, B cells with a few innate cell populations such as ILC3s, type 1 natural killer T (NKT) cells and DC, was captured by this analysis and explained the disease clustering (figure 2D). Noteworthy, multidimensional scaling representation also confirmed the good separation of the five identified clusters of diseases (online supplemental figure S2).

### The ICOS+/LAG3+ Treg ratio clusters AD and AIF according to their inflammatory status

**Figure 3:**
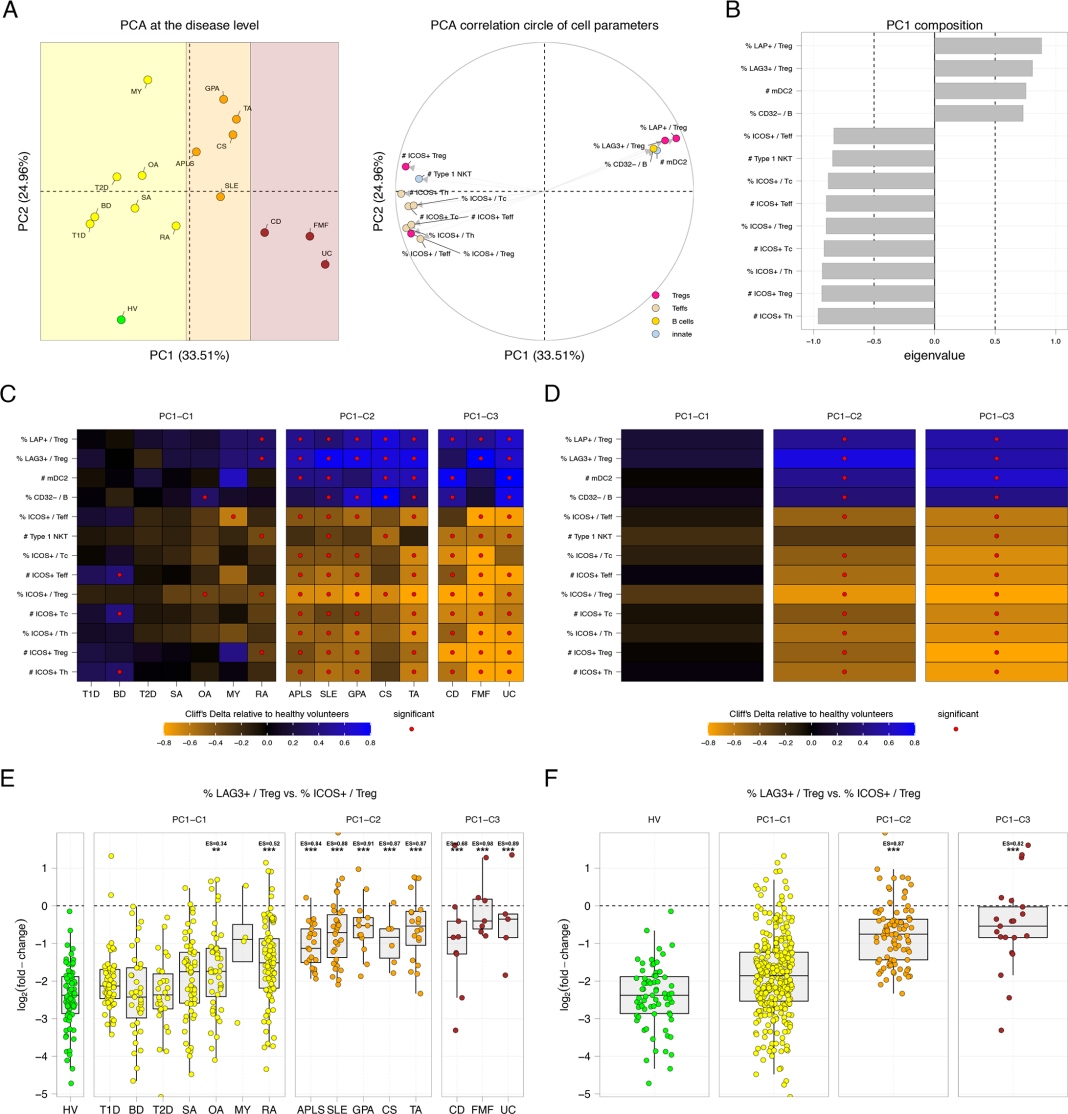
Disorders are spread along a first immunological axis that is mainly driven by an LAG3/ICOS balance in Tregs. (A) Principal component analysis constructed based on Cliff’s Delta values of each group of patients relative to the healthy condition along with the correlation circle of cell parameters exclusively associated with the first principal component (PC1). Conditions are coloured based on the three clusters of diseases identified along the PC1 axis. Selected parameters are coloured based on their immunological families. (B) Bar chart representation showing the eigenvalues of cell parameters exclusively associated with the PC1. Cell parameters with negative eigenvalues are driving the disease on the left of the PCA representation. Cell parameters with positive eigenvalues are driving the disease on the right part of the PCA representation. (C and D) Heatmap representation showing the Cliff’s Delta measures in each disease relative to healthy volunteers for selected cell parameters at the disease or cluster levels. Diseases or clusters of diseases are ordered as projected on the PC1 axis. Parameters significantly dysregulated in one condition are indicated with a red dot. Cell parameters are ordered according to their eigenvalues. (E and F) Boxplot and jitter representations showing the log_2_ fold change between the percentage of ICOS+ cells within Tregs and the percentage of LAG3+ cells within Tregs at the disease or cluster disease levels. Significant comparisons to healthy volunteers are indicated with their p values (**p<0.01, ***p<0.001) and Cliff’s Delta effect size (ES) measure. APLS, antiphospholipid syndrome; BD, Behçet’s disease; CD, Crohn’s disease; CS, Churg-Strauss disease; FMF, familial Mediterranean fever; GPA, granulomatosis with polyangiitis; HV, healthy volunteer; MY, myositis; OA, osteoarthritis; PCA, principal component analysis; RA, rheumatoid arthritis; SA, spondyloarthritis; SLE, systemic lupus erythematosus; T1D, type 1 diabetes; T2D, type 2 diabetes; TA, Takayasu arteritis; UC, ulcerative colitis.

We determined that disorders were spread into three clusters along the first axis (PC1) of the PCA (figure 3A). This clustering appears to be associated with the disease inflammatory levels. The first cluster (PC1-C1) comprised HV, T1D and T2D that are associated with no inflammation. Additionally, this cluster contained BD, SA, OA, MY and RA diseases that are disorders with low inflammatory levels. The second cluster (PC1-C2) comprised APLS, GPA, CS and TA diseases that are disorders with moderate inflammatory levels. Finally, the third cluster (PC1-C3) comprised CD, FMF and UC that are disorders with high inflammatory levels. Of note, SLE was classified in the second cluster (PC1-C2) associated with moderate inflammatory levels.

This first axis was specifically associated with 13 cell parameters (figure 3A,B). Four parameters positively correlated with this PC1 axis—and drove diseases on the right part of the PCA. These parameters consisted of the percentages of LAP+ cells among Tregs, LAG3+ cells among Tregs, CD32− cells among B cells and the absolute number of mDC2. Nine parameters negatively correlated with this PC1 axis and drove diseases on the left part of the PCA. These parameters consisted of multiple cell parameters associated with ICOS+ in Tregs, Th or Tc, as well as the number of type 1 NKT cells.

Heatmap representations of effect size measures for these 13 cell parameters showed a clear gradient of downregulation or upregulation relative to HV along this first axis in the three clusters (figure 3C,D). This set of 13 parameters was almost not significantly impacted in the first cluster (PC1-C1) and was increasingly significantly impacted in the second and the third clusters (PC1-C2 and PC1-C3). The absolute numbers of ICOS+ Tregs and ICOS+ Teffs, and the percentage of LAG3+ cells among Tregs were representative of this gradient (online supplemental figure S3A–C). Of note, we found a strong positive correlation between the absolute numbers of ICOS+ Teffs and ICOS+ Tregs (online supplemental figure S3D).

Furthermore, we identified that the balance between the percentages of LAG3+ Tregs and ICOS+ Tregs was the most determining factor of this gradient (figure 3E,F).

### Activated Treg subsets and ILC3 clusters AD and AIF according to the type of affected tissues

**Figure 4:**
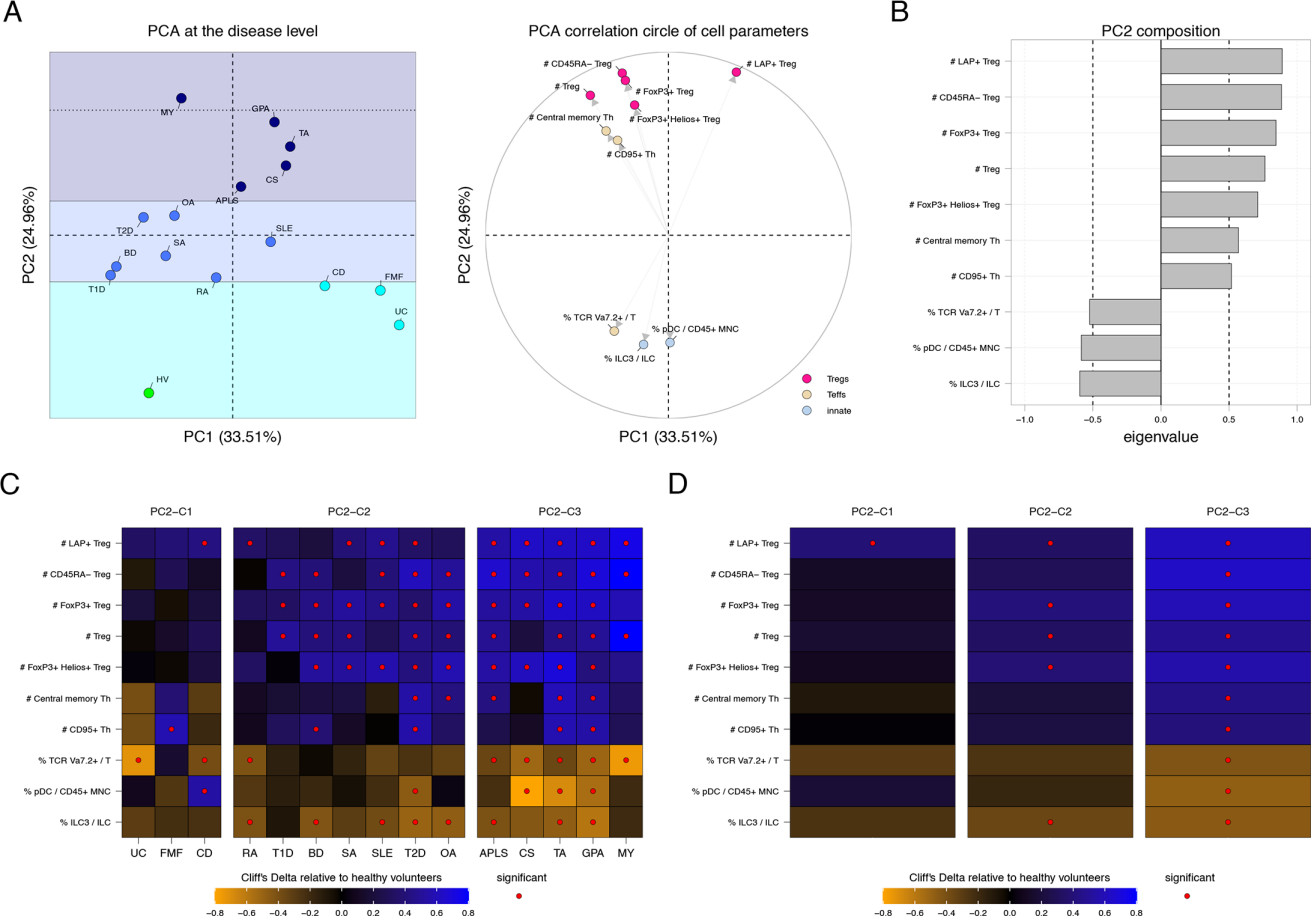
Disorders are spread along a second immunological axis that is mainly driven by Treg subsets and type 3 ILC. (A) Principal component analysis constructed based on Cliff’s Delta values of each group of patients relative to the healthy condition along with the correlation circle of cell parameters exclusively associated with the second principal component (PC2). Conditions are coloured based on the three clusters of disease identified along the PC2 axis. Selected parameters are coloured based on their immunological families. (B) Bar chart representation showing the eigenvalues of cell parameters exclusively associated with the PC2. Cell parameters with negative eigenvalues are driving the disease at the bottom of the PCA representation. Cell parameters with positive eigenvalues are driving the disease on the top part of the PCA representation. (C and D) Heatmap representation showing the Cliff’s Delta measures in each disease relative to healthy volunteers for all cell parameters at the disease or cluster levels. Diseases or clusters of diseases are ordered as projected on the PC2 axis. Parameters significantly dysregulated in one condition are indicated with a red dot. Cell parameters are ordered according to their eigenvalues. APLS, antiphospholipid syndrome; BD, Behçet’s disease; CD, Crohn’s disease; CS, Churg-Strauss disease; FMF, familial Mediterranean fever; GPA, granulomatosis with polyangiitis; HV, healthy volunteer; ILC, innate lymphoid cell; MY, myositis; OA, osteoarthritis; PCA, principal component analysis; RA, rheumatoid arthritis; SA, spondyloarthritis; SLE, systemic lupus erythematosus; T1D, type 1 diabetes; T2D, type 2 diabetes; TA, Takayasu arteritis; UC, ulcerative colitis.

We identified that disorders were spread into three clusters along the second axis (PC2) of the PCA (figure 4A). UC, CD and FMF diseases (that are IBD and IBD-like disorders) were localised on the bottom of the PC2 axis, in a first cluster (PC2-C1). RA, BD, SA, SLE and OA (that are arthritis disorders) as well as T1D and T2D (that are metabolic diseases) were positioned in the middle of the PC2 axis, in a second cluster (PC2-C2). Finally, APLS, CS, TA, GPA (that are blood vessel disorders) and MY diseases were located at the top of the PC2 axis, in a C3 disease cluster, with MY (that is a muscle disorder) being at the extreme top of the PCA (PC2-C3). Thus, PC2 appears to cluster diseases based on affected tissues.

This second axis was specifically associated with 10 cell parameters (figure 4A,B). Seven parameters positively correlated with this PC2 axis, driving the diseases to the top of the PCA. These cell parameters consisted of Tregs and Treg subsets—including LAP+ Tregs, CD45RA− Tregs and FoxP3+ Tregs—central memory CD4+ T cells and CD95+ Th cells. Conversely, three cell populations negatively correlated with this PC2 axis driving the diseases on the bottom of the PCA representation. These parameters included the percentage of TCR Va7.2+ cells among T cells, the percentage of plasmacytoid dendritic cells among CD45+ mononuclear cells and the percentage of ILC3s among ILCs.

Heatmap representations of effect size measures for this set of 10 cell parameters showed a clear gradient of regulations relative to HV along this second axis in the three clusters (figure 4C,D). This set of 10 parameters was almost not significantly impacted in the PC2-C1 cluster and was increasingly significantly impacted in the PC2-C2 and PC2-C3 clusters. The percentages of LAP+ cells among Tregs, the absolute number of LAP+ Tregs, the percentage of ILC3s among ILCs and the absolute number of ILC3s were representative of this gradient of dysregulations (online supplemental figure S4).

### Disease clusters can be captured by a restricted core of immune cell populations

**Figure 5:**
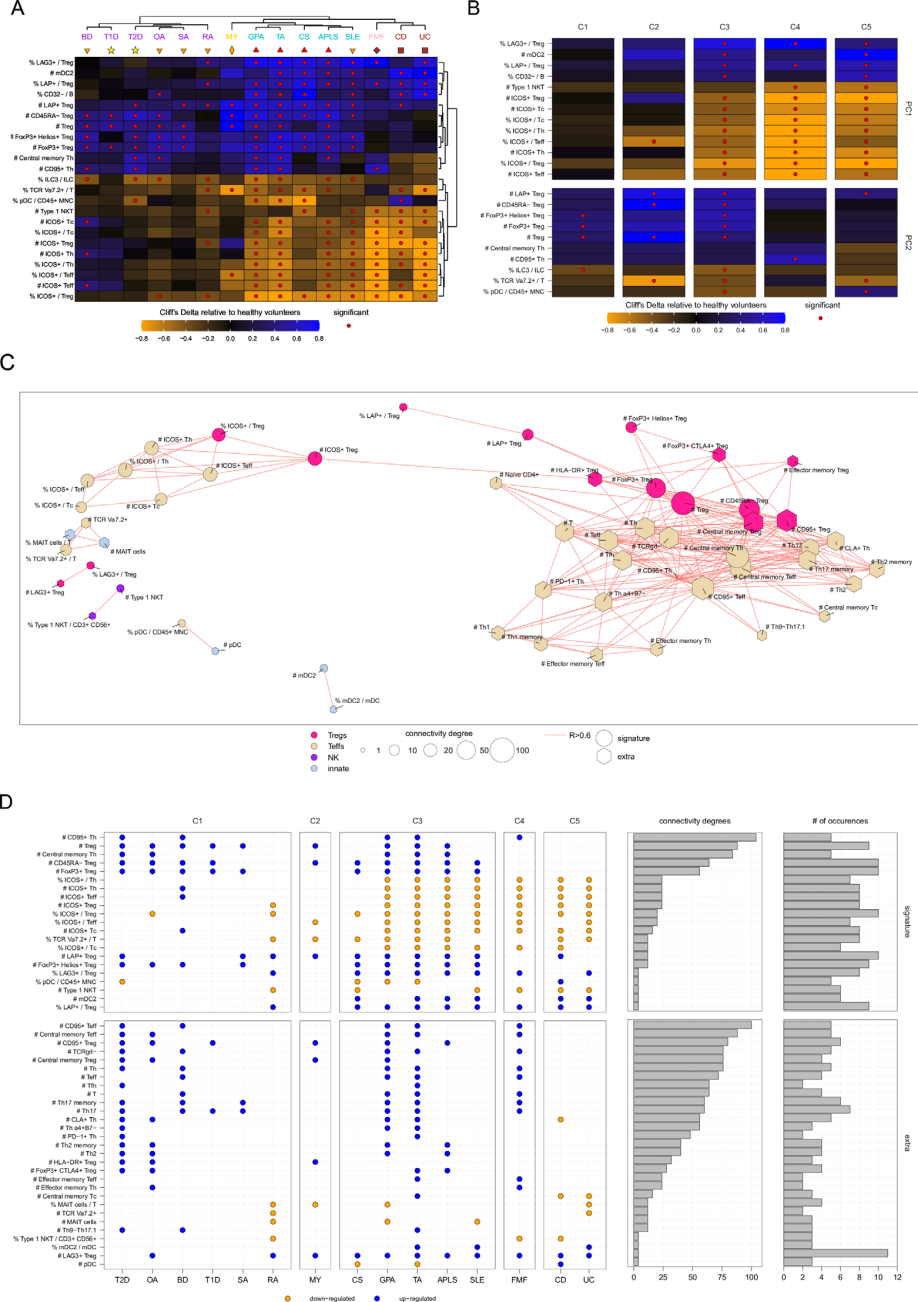
Disease clusters can be captured by a restricted core of immune cell populations. (A) Heatmap representation of Cliff’s Delta effect size measurements relative to healthy volunteers in all diseases for the 23 cell parameters associated with first principal component (PC1) and PC2. Unsupervised hierarchical clustering was used to automatically gather diseases and cell populations. Diseases are coloured based on their associated inflammation levels (no-inflammation levels in yellow, low-inflammation levels in orange, moderate-inflammation levels in red, high-inflammation levels in brown) and shaped based on their type/localisation (stars for metabolic diseases, down triangles for arthritis, up triangles for blood vessels, squares for inflammatory bowel disease (IBD) and lozenge for IBD-like). (B) Heatmap representation of Cliff’s Delta effect size measurements relative to healthy volunteers in the five clusters of diseases for the 23 cell parameters associated with PC1 and PC2. (C) Coexpression network showing the significant correlations between cell parameters and other cell parameters. Parameters included in the immunological signature are represented with circles, while other cell parameters associated with parameters of this signature are represented with hexagonal shapes. Parameters are coloured according to their immunological families. (D) Dot plot and bar plot representations showing cell parameters of the signature or associated with the signature statistically different in diseases relative to healthy volunteers along with their connectivity degrees in the coexpression network and their number of occurrences across diseases. APLS, antiphospholipid syndrome; BD, Behçet’s disease; CD, Crohn’s disease; CS, Churg-Strauss disease; FMF, familial Mediterranean fever; GPA, granulomatosis with polyangiitis; ILC, innate lymphoid cell; MY, myositis; OA, osteoarthritis; RA, rheumatoid arthritis; SA, spondyloarthritis; SLE, systemic lupus erythematosus; T1D, type 1 diabetes; T2D, type 2 diabetes; TA, Takayasu arteritis; UC, ulcerative colitis.

Together, the signature of 23 cell parameters significantly associated with PC1 or PC2 axes was enough to capture the five clusters of diseases that were previously identified (figure 5A). The C1 disease cluster contained T1D and T2D—which are two metabolic disorders without inflammation—along with BD, OA, RA and SA—which are arthritis disorders with low inflammation degree. The C2 disease cluster contained MY alone—a muscle disorder with a low inflammation. The C3 disease cluster contained APLS, CS, GPA and TA—which are blood vessel disorders with moderate degrees of inflammation. Finally, the C4 and C5 disease clusters comprised CD, FMF and UC—which are inflammatory bowel or inflammatory bowel-like diseases with high degrees of inflammation. Of note, SLE, which is mainly an arthritis disease generally associated with low inflammation degree, was found to belong to the C3 disease cluster.

Each cluster of disease was characterised by specific patterns of downregulated or upregulated cell parameters relative to the healthy group along the PCA axes (figure 5B). The C1 disease cluster was characterised by no dysregulations of parameters associated with the PC1 axis and limited significant dysregulations of parameters associated with the PC2 axis. The C2 disease cluster, which comprised MY alone, was characterised by dysregulation of cell parameters mainly associated with the PC2 axis. The third clustering of diseases was characterised by significant dysregulation of cell parameters associated with both PC1 and PC2 axes. Finally, the C4 and C5 disease clusters were characterised by dysregulations of cell parameters mainly associated with the PC1 axis.

To further investigate the characteristics of this signature, we created a coexpression network based on its parameters (figure 5C). We identified a main community of Teff− and Treg− parameters positively correlated. Among them, the number of CD95+ Th/Teffs/Tregs, the number of Tregs and the number of central memories Th/Teff had the highest connectivity degrees, reflecting potential central activities in immune dysregulations and could serve as biomarkers (figure 5D).

### The number of LAG3+ Tregs is a key immunological marker in multiple ADs and AIFs

To complement our findings, we generated classification trees capturing parameters or combination of parameters that best separate patients from healthy individuals in each disease (online supplemental figures S5–S7). These supervised analyses aimed to capture the most discriminating features in each disease independently. Each classification tree was generated using all available cell parameters, and we quantified the model classification precisions.

**Figure 6:**
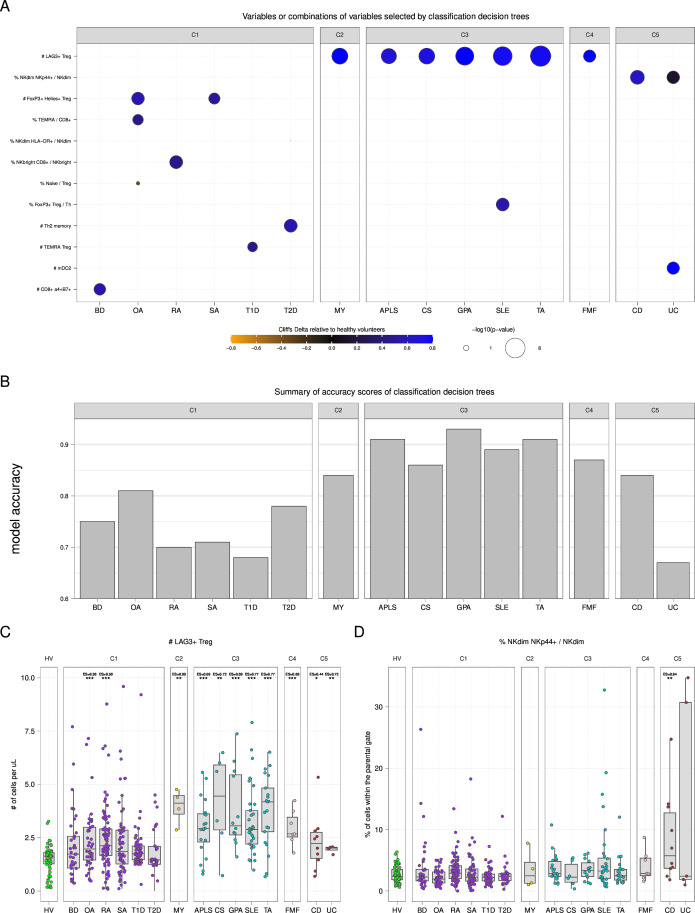
Classification decision trees identify LAG3+ Tregs as a key marker in multiple diseases. (A) Dot plot representation showing cell parameters found to be significantly involved in classification decision trees. For each disease, a classification decision tree was generated to identify the best marker or combination of markers that distinguish patients from healthy volunteers. The size of each dot is proportional to the −log10 of the p value, and dots are coloured according to the effect size relative to healthy volunteers. Cell parameters are sorted based on their number of occurrences in the generated models. (B) Accuracy, ranging from 0 to 1, of each classification decision tree are represented using a bar chart representation. (C and D) Boxplot and jitter representations showing the number of LAG3+ Tregs and the percentage of NKp44+ within NK^dim^ cells for each disease. Diseases are gathered by identified disease clusters. Significant comparisons to healthy volunteers are indicated with their p values (*p<0.05, **p<0.01, ***p<0.001) and Cliff’s Delta effect size (ES) measure. APLS, antiphospholipid syndrome; BD, Behçet’s disease; CD, Crohn’s disease; CS, Churg-Strauss disease; FMF, familial Mediterranean fever; GPA, granulomatosis with polyangiitis; HV, healthy volunteer; MY, myositis; OA, osteoarthritis; RA, rheumatoid arthritis; SA, spondyloarthritis; SLE, systemic lupus erythematosus; T1D, type 1 diabetes; T2D, type 2 diabetes; TA, Takayasu arteritis; UC, ulcerative colitis.

A total of 12 cell parameters were found by classification trees (figure 6A,B). We found that patients from the C1 disease cluster were characterised by heterogeneous disease-specific markers rather than a common set of markers. The p values associated with the selected parameters were less significant, and the accuracy of decision tree predictions was lower for these diseases compared with other clusters of diseases. Patients from C2, C3 and C4 disease clusters were all characterised by a higher number of LAG3+ Tregs compared with HVs, with more significant p values and higher decision tree predictions compared with other clusters of diseases. Finally, the C5 disease cluster was characterised by a higher percentage of NKp44+ cells among NK^dim^ cells relative to HV. Of note, the downregulation of the number of ICOS+ Tregs was found to be the most discriminative parameter to segregate all patients—regardless of their diseases—from controls, with a limited classification precision (online supplemental figure S8).

Of note, the absolute number of LAG3+ Tregs and the abundance of NKp44+ cells among NK^dim^—that were statistically different from healthy donors in clusters C2, C3, C4 and C5—were heterogeneous within diseases. This suggests that these parameters could be potential markers for subsets of patients belonging to clusters C2, C3, C4 and C5 (figure 6C,D).

Together, these independent supervised analyses confirmed the role of LAG3+ if Treg cells as a key immunological marker in multiple ADs and AIFs, along with ICOS+ Tregs to a lesser extent.

## Discussion

We aimed to use deep immunophenotyping of blood samples drawn from a diverse cohort of patients suffering from various ADs and AIFs,^10^ to describe and classify better AIF and AD.

### A proposed classification of AD and AIF using deep immunophenotyping

Based on 15 disorders ranging from purely autoimmune to purely inflammatory, we have identified five clusters of diseases, proposing a classification of these diseases based on deep immunophenotyping profiles. Noteworthily, the robustness of this clustering was shown using three unsupervised analytical approaches: hierarchical clustering, PCA and multidimensional scaling.

Each cluster of disease was characterised by specific patterns of downregulated or upregulated cell parameters relative to the healthy group. Overall, BD, OA, RA, SA, T1D and T2D, all grouped in the C1 disease cluster, exhibited an immunological profile that did not markedly deviate from that of HV. Conversely, diseases classified in the other clusters demonstrated a distinct divergence from HV.

We identified a core signature of 23 markers able to capture the proposed disease clustering. We found that CD95+ Th/Teff/Treg were associated with this signature, upregulated relative to HV. CD95 is a cell surface receptor that plays a pivotal role in regulating apoptosis or programmed cell death.^18^ Mutation or dysregulation of the CD95 apoptotic pathway can be involved in various diseases, including AD.^18 19^ We hypothesise that continuous exposure to self-antigens can keep these T cells activated, leading to increased CD95 expression as a part of the feedback mechanism to regulate excessive immune responses.

A predominant characteristic of most of the diseases included in this study was an augmented count of LAG3+ Tregs, as demonstrated by the supervised classification trees. LAG3 is an immune checkpoint that binds to Major Histocompatibility Complex class II (MHCII), and which main role appears to be the inhibition of T cell activation. LAG3 plays various and sometimes controversial roles in autoimmunity, tumour immunity and anti-infection immunity.^20–22^ However, the distinct function of LAG3 on Tregs is largely unknown.

### The LAG3+/ICOS+ balance in Tregs is associated with disease inflammation levels

We identified a first immunological axis, mainly characterised by a LAG3+/ICOS+ balance in Tregs, that is associated with disease inflammation levels. Of note, our analysis positioned SLE in the middle of this inflammation axis, consistent with the observation made by El-Shebiny and colleagues emphasising that SLE is one of the best examples of bridging between AIF and AD.^23^ Indeed, SLE is characterised by a secretion of autoantibodies but also as an interferonopathy.^24^ Of note, only five out of our 33 patients with SLE were diagnosed with coexisting secondary APLS, which limits the contribution of this secondary diagnosis to our observation. In this line, while different studies have pointed out the bivalent characteristics of SA with both autoinflammatory and autoimmune factors,^25 26^ our results highlight a limited dysregulation of cell parameters associated with the inflammation axis in blood.

The percentage of LAG3+ cells among Tregs was directly associated with this immunological axis associated with the inflammation degree of disorders. These results are consistent with the model proposed by Zhang and colleagues, suggesting that the LAG3 intrinsically limits Treg proliferation and functionality by repressing pathways that promote the maintenance of Treg cells at inflammatory sites.^27^ An alternative explanation is that this overexpression of LAG3 in Tregs could reflect an immune exhaustion associated with the disease inflammation degrees. Indeed, overexpression of inhibitory receptors is a characteristic of exhausted T cells.^28^ Our results warrant further examination of the role of LAG3+ Tregs in AD and AIF.

We observed a downregulation of ICOS+ cells within both Tregs and Teffs. Downregulation of ICOS in Teffs and Tregs can have several implications. First, ICOS is an important costimulatory molecule that enhances T cell activation.^29^ Its downregulation can thus potentially impair the activation and function of T cells, leading to a weakened immune response. Second, ICOS is crucial for the function of Tregs, which play a key role in maintaining immune tolerance and preventing autoimmunity.^30^ Downregulation of ICOS in Tregs can disrupt their suppressive functions, potentially leading to an increased risk of AD. Third, ICOS stimulation is known to promote the production of certain cytokines by T cells, such as interleukin 10 (IL-10) and IL-4.^31^ Therefore, ICOS downregulation may lead to decreased cytokine production, potentially affecting immune responses. Fourth, ICOS could have an impact on antibody responses as it plays a role in B cell activation and germinal centre responses.^32^ Of note, the strong correlation observed between ICOS+ Teffs and ICOS+ Tregs suggests an interdependence or cross-regulation between these two subpopulations of T lymphocytes.

Our results show that the increased number of FoxP3+ Helios+ Tregs and the percentage of T Effector Memory RA (TEMRA) among CD8+ were the most discriminant cell parameters to distinguish patients with OA from HVs. Additionally, the increased number of TEMRA Tregs was the most discriminant cell parameter to distinguish patients with T2D from HVs. Although our analysis did not reveal a significant influence of T1D and OA on cell parameters related to the inflammatory axis, we did notice an effect of these diseases on specific cell parameters associated with the disease-type axis, particularly involving ILC3s and activated Treg subsets. Our observations are in line with the notion that even if inflammation is an established secondary component in OA and T2D diseases, autoimmune or autoinflammatory dysregulations could play significant roles in their pathogenesis and in the aetiology of OA^11 12 33^ and T2D.^14 15 34 35^ However, the main discriminating cell parameters in RA and T1D are different from OA and T2D, respectively.

### Activated Tregs and ILC3 are associated with disease types

We identified a second immunological axis, mainly characterised by activated Tregs and ILC3 that is associated with disease types. This axis distinguishes between blood vessel diseases, arthritis and metabolic diseases and IBD/IBD-like diseases. This separation points towards the existence of common immunological mechanisms for each of these groups of diseases and suggests that each of them may require a specifically tailored treatment and management.

While ICOS+ Tregs were downregulated in most diseases, we identified multiple subsets of activated Tregs that were upregulated compared with HV, and that were associated with the disease-type axis. At first, this may be surprising as many ADs have been associated with decreased numbers or proportion of Tregs studied as a whole. We believe that given the fact that Tregs’ main role is to protect us from autoimmunity, developing AD consubstantiality identifies a Treg insufficiency. However, the nature of this insufficiency is likely more complex than usually contemplated and may comprise upregulation of specific subsets that may try to balance the autoimmunity. Actually, multiple groups have made contradictory observations regarding the decrease or increase of Tregs in many ADs and AIFs.^36 37^ Our results highlight the need for more in-depth study of Treg subsets in AD and AIF.

We found that the percentages of ILC3c among ILCs were downregulated in multiple diseases and that this downregulation was associated with the type of diseases. ILCs are predominantly tissue-resident cells, exhibiting remarkable plasticity and adaptability in their functional characteristics.^38^ Several studies have demonstrated the importance of ILC3 in the regulation of tissue homeostasis and their role in regulating inflammatory T cell responses.^39^ ILC3s have been found downregulated in the blood of patients with GPA,^40^ T2D,^41^ SLE^42^ and RA,^43^ relative to healthy controls. ILC3c is implicated in gut homeostasis maintenance and gastrointestinal immune responses.^44^ Furthermore, dysregulation of ILC3 contributes to the progression of IBD.^44 45^ In detail, a dysregulation of NCR− ILC3 or NCR+ ILC3 function and the bias of NCR+ ILC3 towards ILC1 can lead towards these pathogenic conditions.^44^ Clottu *et al* documented the dynamic nature of ILC populations in pathological conditions, observing an increase in ILC1 numbers with a concurrent decrease in ILC3 in the intestines during diseases such as CD.^46^ An interplay between ILC3 and Treg has been reported, especially in the gut and intestine, with ILC3s interacting with microbiota-specific regulatory T cells to establish tolerance in the gut.^47^ Noteworthy, an interplay between ILC3 and LAG+ Tregs has also been reported in the context of gut inflammation.^48 49^ Thus, our results are in line with these observations, with an ILC3 dysregulation being associated with disease type/localisation in AD and AIF.

### A novel nosology of AD and AIF based on disease inflammation degrees and types

In 2006, McGonagle and McDermott proposed a novel classification of AD and AIF, where disorders lay down on a genetically determined gradient ranging from pure AIF to pure AD.^7^ Since, this continuum paradigm has been largely adopted, but also refined.^4 5^ Our results further identified two immunological axes associated with disease inflammation and disease localisation rather than a monodimensional continuum of diseases. We found that the ratio of LAG3+ and ICOS+ in Tregs was associated with disease inflammation levels and that some activated Treg subsets and ILC3s were associated with these types of diseases. This latter observation is in agreement with the fact that tissue-specific factors can serve as components for this classification.^4 7^

### Study limits

A limitation of this study resides in the heterogeneous number of patients across the different disease groups, in large part attributable to the prevalence of diseases. Diseases with larger patient pools can exhibit greater heterogeneity, potentially harbouring subgroups of patients with their own unique immunological specificities. Such a hypothesis might explain why, in diseases with a higher patient count, we identified fewer parameters as significantly impacted. This implies that within these larger groups, individual immune responses may be diverse and not adequately captured by our broad categorisation, potentially masking some specific patterns that could otherwise be informative. On the contrary, the characterisation of diseases with limited numbers of patients in our study, such as FMF (n=7), MY (n=4) and UC (n=5), is not powerful enough statistically and should be taken with moderation. To take into consideration the unbalanced design of our study, we employed the Cliff’s Delta effect size in combination with statistical hypothesis testing to quantify the magnitude and significance of immunological changes. Furthermore, we employed unsupervised and supervised analysis strategies to reduce the potential bias due to the unbalanced design of our study.

Another limitation stems from the heterogeneous degree of disease activity and the diverse treatments received by patients, both of which can impact the immune system. Different stages of a disease can elicit various immune responses, and distinct therapeutic strategies might further modulate these responses. Therefore, while attempting to correlate immune signatures with disease status, we must consider the potential confounding effects of varying disease activity and therapeutic interventions. Nevertheless, it could be considered that the significant parameters revealed by these studies must have a certain robustness as they show up despite this high variability.

Another limitation of this study rests on the cytometry panels that were primarily formulated to target adaptive cell populations. To comprehensively characterise the immune systems of patients with AD and AIF, future steps will necessitate the use of more advanced flow cytometry panels evaluating innate cell populations. The broader and combined analyses of these major components of the immune system should contribute to potentially unveiling novel targets for intervention.

Another limitation of this study is the lack of validation of the proposed clustering on an external dataset. However, to our knowledge, there currently exists no comparable dataset. Specifically, there is a lack of publicly available datasets that screen such an extent of cell parameters collected under similar conditions for a large cohort of patients. This situation significantly limits our ability to perform the necessary external validations. This lack hampers our capacity to conduct the essential external validations. Additionally, the limitation is compounded by the general unavailability of massive deep immunophenotyping data. To mitigate this, we have included the complete set of cell parameters used in our study within the online supplemental materials. This addition aims to serve as a reference for other groups to validate and compare against our identified disease clusters.

## Conclusion

In this study, we outlined the significance of LAG3 and ICOS expression by Tregs, along with various activated Treg subsets and ILCs, in their capacity to act as biomarkers of AIF and AD. These markers offer the means to differentiate diseases based on their type/localisation and their inflammation degrees. Functional studies will now have to investigate the detailed mechanistic roles of these markers and cell populations in various settings. Ultimately, our findings point towards a personalised treatment of patients based on the cell and molecular parameters defining their disease clusters.

## Data Availability

Data are available in a public, open access repository. The relative percentage or absolute count of the 224 cell parameters differentially abundant in at least one disease relative to healthy volunteers is available on the Zenodo open repository through DOI: 10.5281/zenodo.10364382.
