# Supplementary material for: Deep immunophenotyping reveals that autoimmune and autoinflammatory disorders are spread along two immunological axes capturing disease inflammation levels and types
Source: Ann Rheum Dis. 2024 Jan 5;83(5):638–50. doi: 10.1136/ard-2023-225179 (PMC11041612; doi:10.1136/ard-2023-225179)
Supplement: Supplementary data [file ard-2023-225179supp001.pdf]

Supplementary Materials

Condition	Abbreviation	Clinical criteria used for definition
Antiphospholipid syndrome	APLS	Sapporo's criteria [1]
Behçet's disease	BD	International Criteria for Behçet 's disease (ISG/ICBD) [2,3]
Churg-Strauss's disease	CS	American College of Rheumatology (ACR) criteria [4]
Crohn's disease	CD	Clinical and histological features
Familial Mediterranean Fever	FMF	Heller's criteria and *MEFV* mutation [5]
Granulomatosis with polyangiitis	GPA	American College of Rheumatology (ACR) criteria [6]
Myositis	MY	Clinical and biological parameters and grouped into dystrophy, polymyositis, dermatomyositis, inclusion-body myositis, necrotizing and anti-synthetase related myositis [7,8]
Osteoartritis	OA	Kellgren-Lawrence classification of radiographic features [9]
Rheumatoid Arthritis	RA	American College of Rheumatology (ACR) criteria [10] and European League Against Rheumatism (EULAR) criteria [11]
Spondyloarthritis	SA	Assessment of Spondyloarthritis international Society (ASAS) and New York Criteria [12]
Systemic Lupus Erythematosus	SLE	American College of Rheumatology (ACR) criteria [13]
Takayasu arteritis	TA	American College of Rheumatology (ACR) criteria [14]
Type 1 Diabetes	T1D	American Diabetes Association (ADA) criteria [15]
Type 2 Diabetes	T2D	American Diabetes Association (ADA) criteria [16]
Ulcerative Colitis	UC	Clinical and histological features

Table S1 – Clinical criteria used for disease diagnostics. For each disease, the clinical criteria utilized for its definition are detailed, accompanied by corresponding literature references (as supplemental references).

	APLS (n=23)	AS (n=58)	BD (n=38)	CD (n=10)	CS (n=6)	FMF (n=7)	GPA (n=14)	MY (n=4)	OA (n=45)	RA (n=91)	SLE (n=33)	T1D (n=60)	T2D (n=27)	TA (n=22)	UC (n=5)
csDMARDs	n=4 (17.39%)	n=9 (15.52%)	n=15 (39.47%)	n=7 (70.00%)	n=2 (33.33%)	n=0 (0.00%)	n=6 (42.86%)	n=3 (75.00%)	n=1 (2.22%)	n=67 (73.62%)	n=31 (93.94%)	n=1 (1.67%)	n=0 (0.00%)	n=9 (40.91%)	n=4 (80.00%)
csDMARDs:: immunosuppressants	n=1 (4.34%)	n=6 (10.34%)	n=15 (39.47%)	n=7 (70.00%)	n=2 (33.33%)	n=0 (0.00%)	n=6 (42.86%)	n=3 (75.00%)	n=0 (0.00%)	n=64 (70.32%)	n=13 (39.39%)	n=1 (1.67%)	n=0 (0.00%)	n=9 (40.91%)	n=3 (60.00%)
csDMARDs:: immunosuppressants:: Mmethotrexate	n=1 (4.34%)	n=5 (8.62%)	n=4 (10.53%)	n=0 (0.00%)	n=1 (16.67%)	n=0 (0.00%)	n=3 (21.43%)	n=0 (0.00%)	n=0 (0.00%)	n=54 (59.34%)	n=3 (9.09%)	n=0 (0.00%)	n=0 (0.00%)	n=9 (40.91%)	n=0 (0.00%)
csDMARD:: Hydroxychloroquine	n=4 (17.39%)	n=0 (0.00%)	n=0 (0.00%)	n=0 (0.00%)	n=0 (0.00%)	n=0 (0.00%)	n=0 (0.00%)	n=0 (0.00%)	n=1 (2.22%)	n=5 (5.49%)	n=28 (84.85%)	n=0 (0.00%)	n=0 (0.00%)	n=0 (0.00%)	n=0 (0.00%)
Oral glucocorticoids	n=4 (17.39%)	n=9 (15.52%)	n=22 (57.89%)	n=4 (40.00%)	n=5 (83.33%)	n=1 (14.29%)	n=12 (85.71%)	n=2 (50.00%)	n=3 (6.67%)	n=53 (58.24%)	n=20 (60.61%)	n=1 (1.67%)	n=2 (7.41%)	n=16 (72.73%)	n=2 (40.00%)
bDMARDs	n=0 (0.00%)	n=4 (6.90%)	n=16 (42.11%)	n=4 (40.00%)	n=0 (0.00%)	n=0 (0.00%)	n=6 (42.86%)	n=0 (0.00%)	n=0 (0.00%)	n=19 (20.87%)	n=7 (21.21%)	n=1 (1.67%)	n=0 (0.00%)	n=9 (40.91%)	n=2 (40.00%)
bDMARDs:: TNF inibitors	n=0 (0.00%)	n=2 (3.45%)	n=8 (21.05%)	n=4 (40.00%)	n=0 (0.00%)	n=0 (0.00%)	n=0 (0.00%)	n=0 (0.00%)	n=0 (0.00%)	n=12 (13.18%)	n=0 (0.00%)	n=0 (0.00%)	n=0 (0.00%)	n=3 (13.64%)	n=1 (20.00%)
Paracetamol	n=5 (21.73%)	n=17 (29.31%)	n=8 (21.05%)	n=0 (0.00%)	n=0 (0.00%)	n=3 (42.86%)	n=5 (35.71%)	n=1 (25.00%)	n=23 (51.11%)	n=42 (46.15%)	n=5 (15.15%)	n=1 (1.67%)	n=3 (11.11%)	n=6 (27.27%)	n=0 (0.00%)
NSAIDs	n=2 (8.69%)	n=35 (60.34%)	n=7 (18.42%)	n=0 (0.00%)	n=0 (0.00%)	n=0 (0.00%)	n=0 (0.00%)	n=0 (0.00%)	n=13 (28.89%)	n=18 (19.78%)	n=3 (9.09%)	n=1 (1.67%)	n=0 (0.00%)	n=2 (9.09%)	n=1 (20.00%)
Insulin	n=0 (0.00%)	n=0 (0.00%)	n=1 (2.63%)	n=0 (0.00%)	n=0 (0.00%)	n=0 (0.00%)	n=1 (7.14%)	n=0 (0.00%)	n=0 (0.00%)	n=1 (1.09%)	n=1 (3.03%)	n=56 (93.33%)	n=5 (18.52%)	n=3 (13.64%)	n=0 (0.00%)
Oral antidiabetics	n=1 (4.34%)	n=1 (1.72%)	n=0 (0.00%)	n=0 (0.00%)	n=0 (0.00%)	n=0 (0.00%)	n=2 (14.29%)	n=0 (0.00%)	n=2 (4.44%)	n=0 (0.00%)	n=2 (6.06%)	n=6 (10.00%)	n=25 (92.59%)	n=3 (13.64%)	n=0 (0.00%)

Table S2 – Main treatments given to groups of patients. For each disease, the main treatments provided to patients are indicated in numbers and percentage of patients with the associated disease. csDMARDs stands for conventional synthetic Disease-Modifying Antirheumatic Drugs. bDMARD stands for biological Disease-Modifying Antirheumatic Drugs. NSAIDs stands for Non-Steroidal Antinflammatory Drugs.

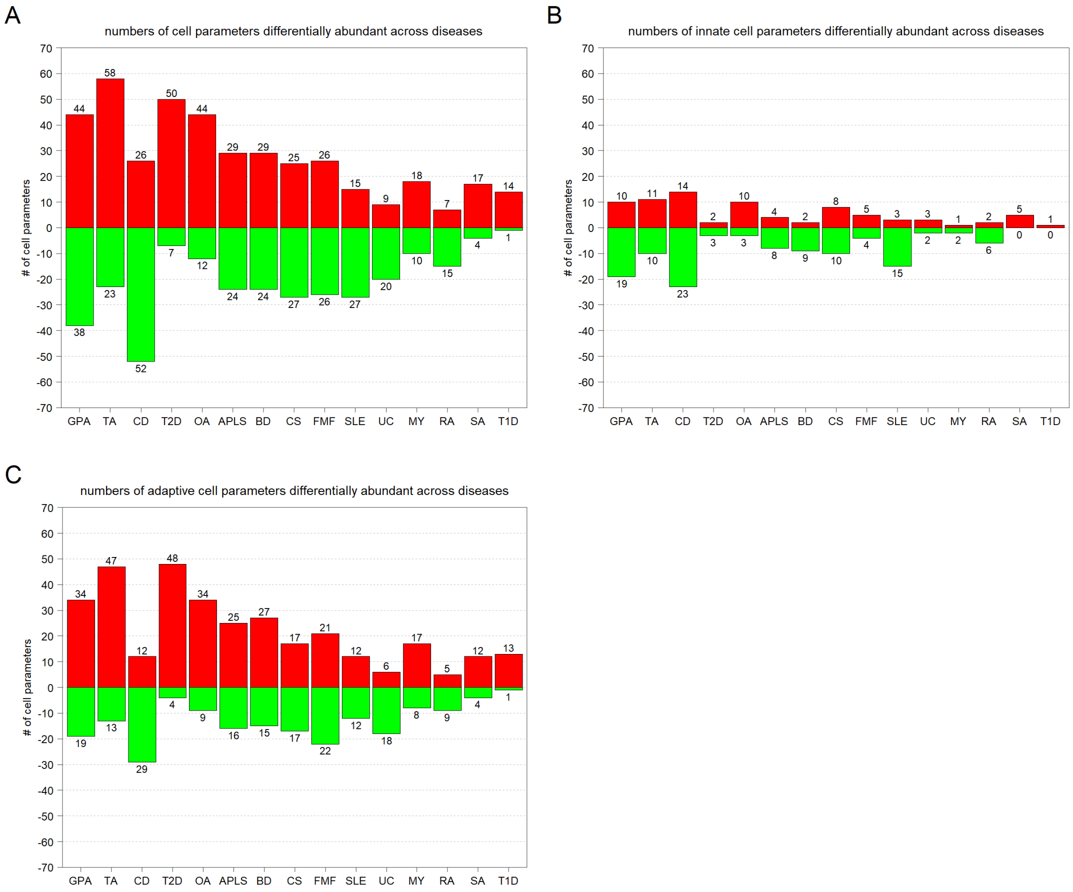

Figure S1. Bar chart representation showing the number of immune (**A**), innate (**B**), and adaptive (**C**) cell parameters differentially abundant in each disease relative to healthy volunteers. The numbers of up-regulated cell parameters are indicated in red, and the numbers of down-regulated cell parameters are indicated in green. Diseases are ordered based on their total numbers of down- or up- regulated cell parameters relative to the healthy condition.

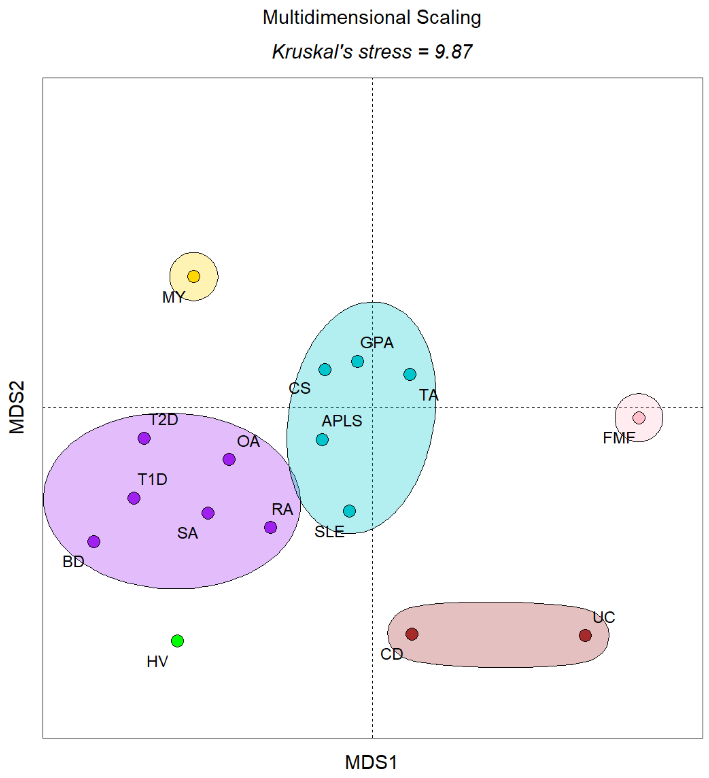

Figure S2. Multidimensional scaling representation generated based on Cliff's Delta values of selected cell parameters in each disease compared to healthy volunteers. Each dot corresponds to a disease, and dots are positioned in a two-dimensional space based on their similarities computed using Cliff's Delta values. Diseases are colored based on the 5 disease clusters identified by unsupervised hierarchical clustering. The Kruskal Stress indicates the quality of the representation and is proportional to the amount of information lost during the dimensionality reduction process.

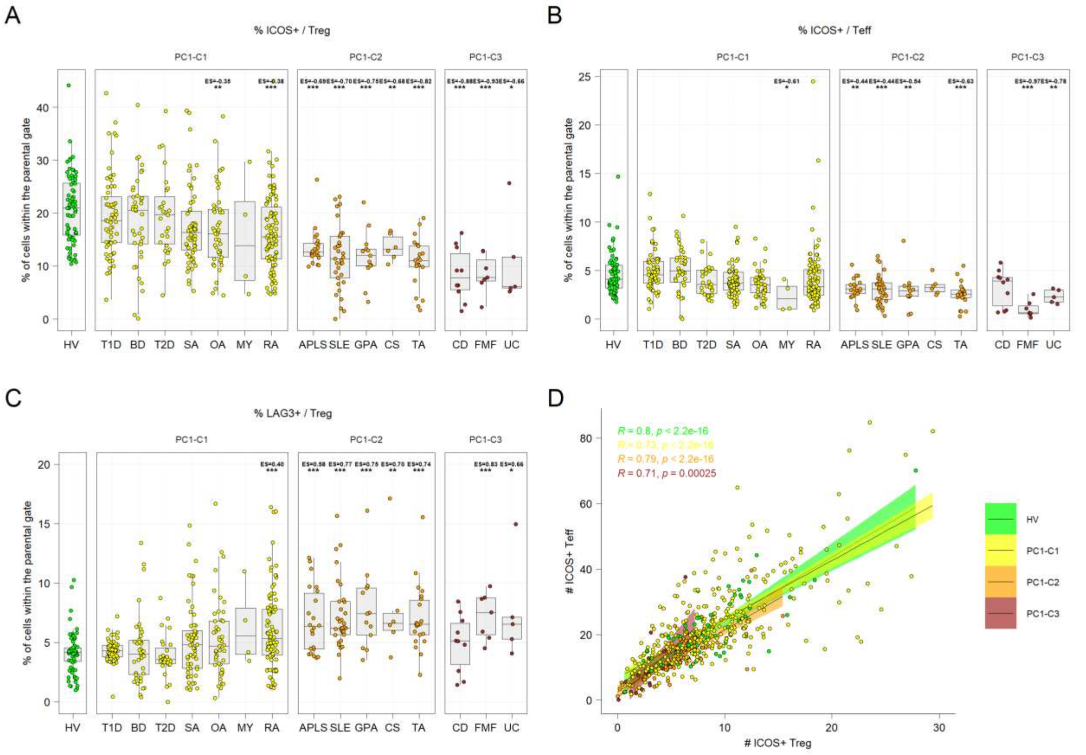

Figure S3. (**A, B,** and **C**) Boxplot and jitter representations showing the percentage of ICOS$^+$ cells within Tregs and Teffs as well as the percentage of LAG3$^+$ cells among Tregs. Significant comparisons to healthy volunteers are indicated with their p-values (*: p-value<0.05, **: p-value<0.01, ***: p-value<0.001) and Delta's Cliff Effect Size measure (ES). (**D**) Scatter plot representation showing the Spearman correlation between the absolute numbers of ICOS+ Tregs and ICOS+ Teffs within each cluster of disease identified along the PC1.

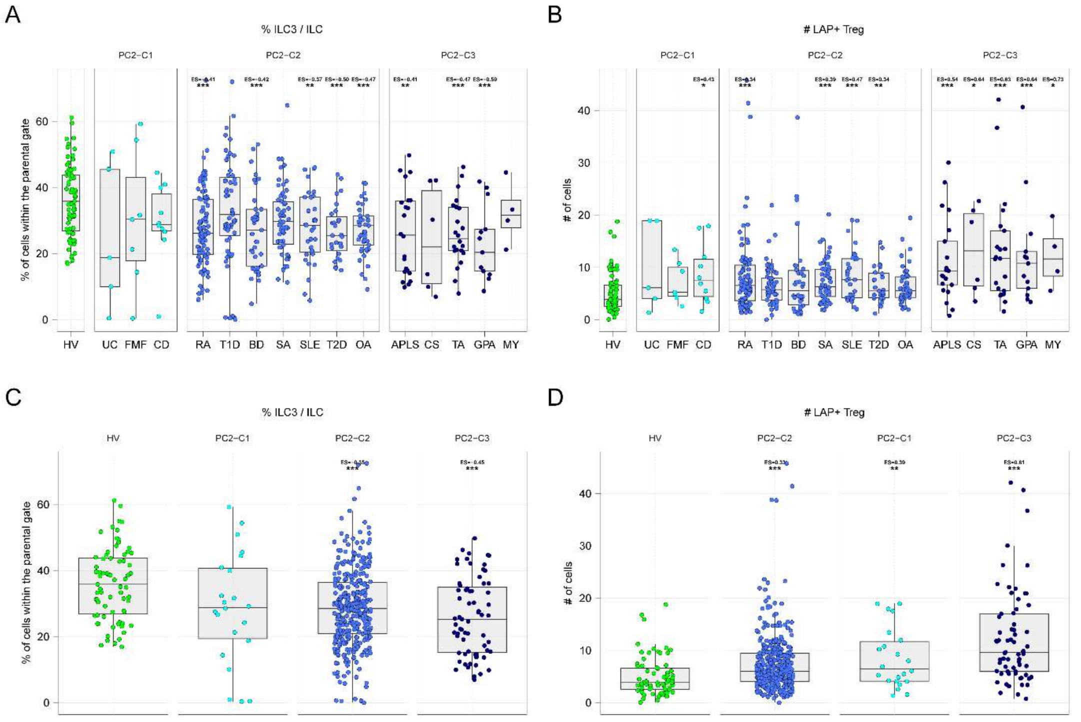

Figure S4. Boxplot and jitter representations showing the percentages of ILC3s among ILCs and the absolute numbers of LAP3+ Tregs at the disease (**A** and **B**) and cluster (**C** and **D**) levels. Significant comparisons to healthy volunteers are indicated with their p-values (*: p-value<0.05, **: p-value<0.01, ***: p-value<0.001) and Delta's Cliff Effect Size measure (ES).

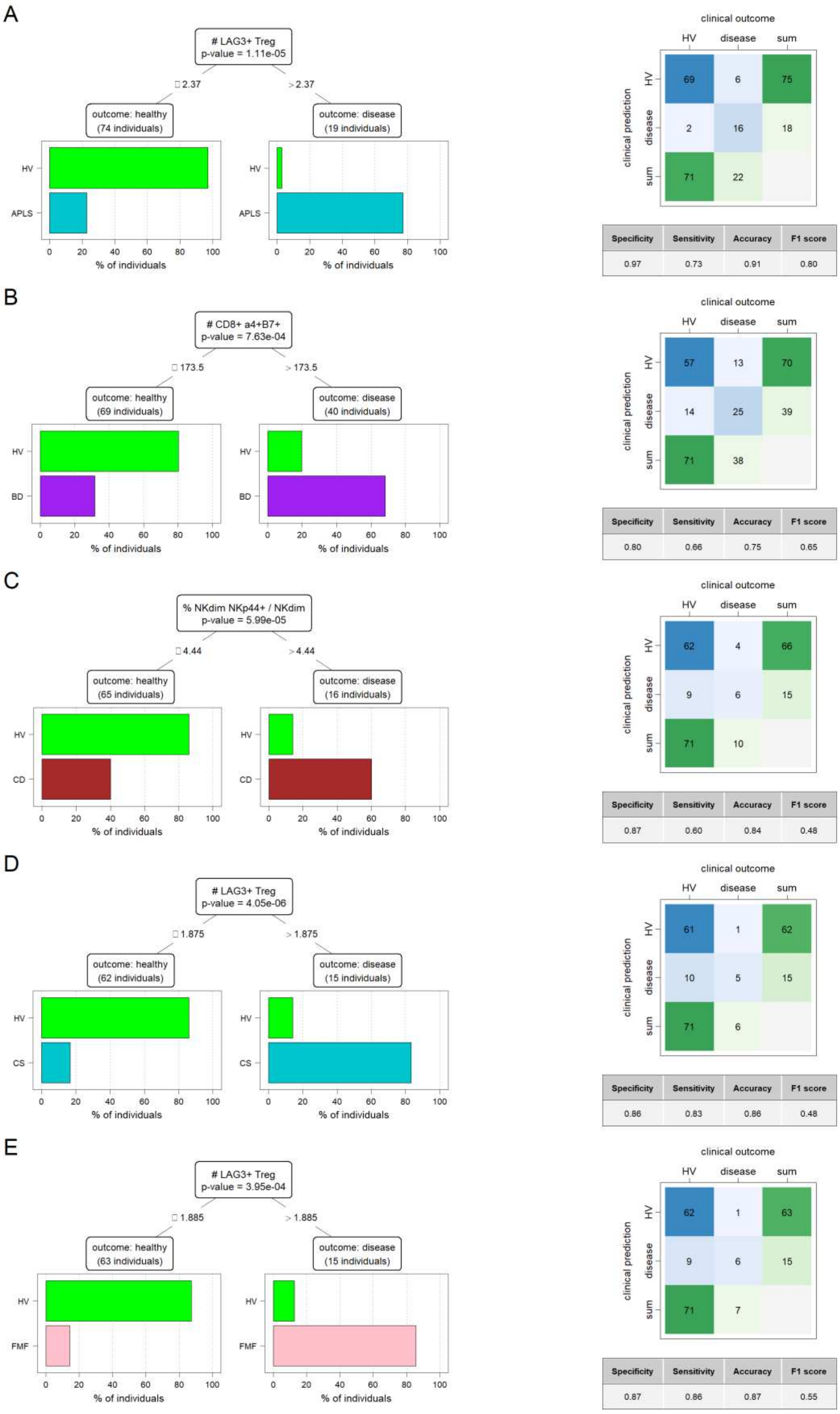

Figure S5. (**A-E**) Classification decision trees constructed to discriminate APLS, BD, CD, CS, FMF patients from healthy volunteers. For each classification leaf, the percentages of well-classified patients and well-classified individuals are indicated. The specificity, sensitivity, accuracy, and F1 score measures associated with each model are indicated along with the confusion matrix.

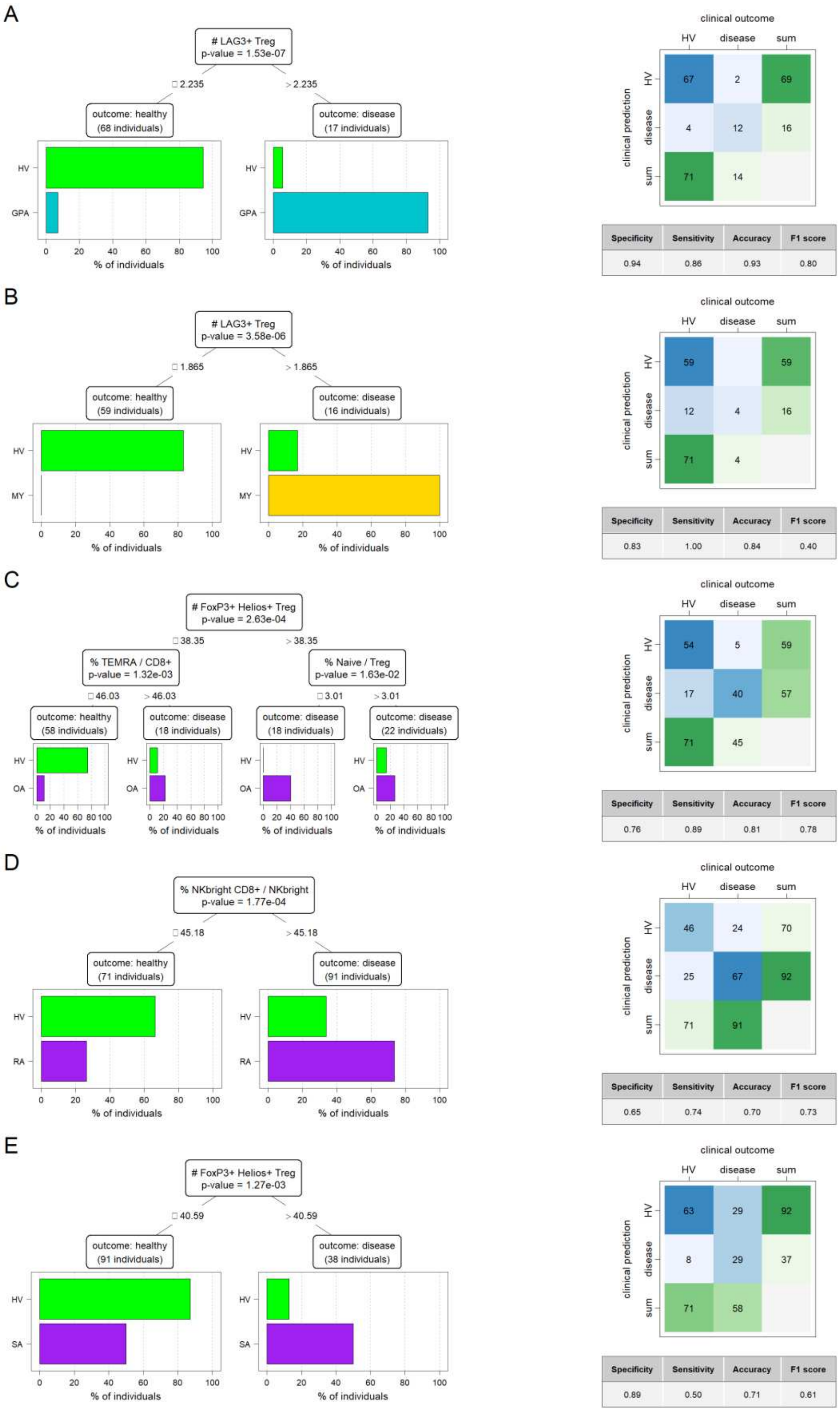

Figure S6. (**A-E**) Classification decision trees constructed to discriminate GPA, MY, OA, RA, and SA patients from healthy volunteers. For each classification leaf, the percentages of well-classified patients and well-classified individuals are indicated. The specificity, sensitivity, accuracy, and F1 score measures associated with each model are indicated along with the confusion matrix.

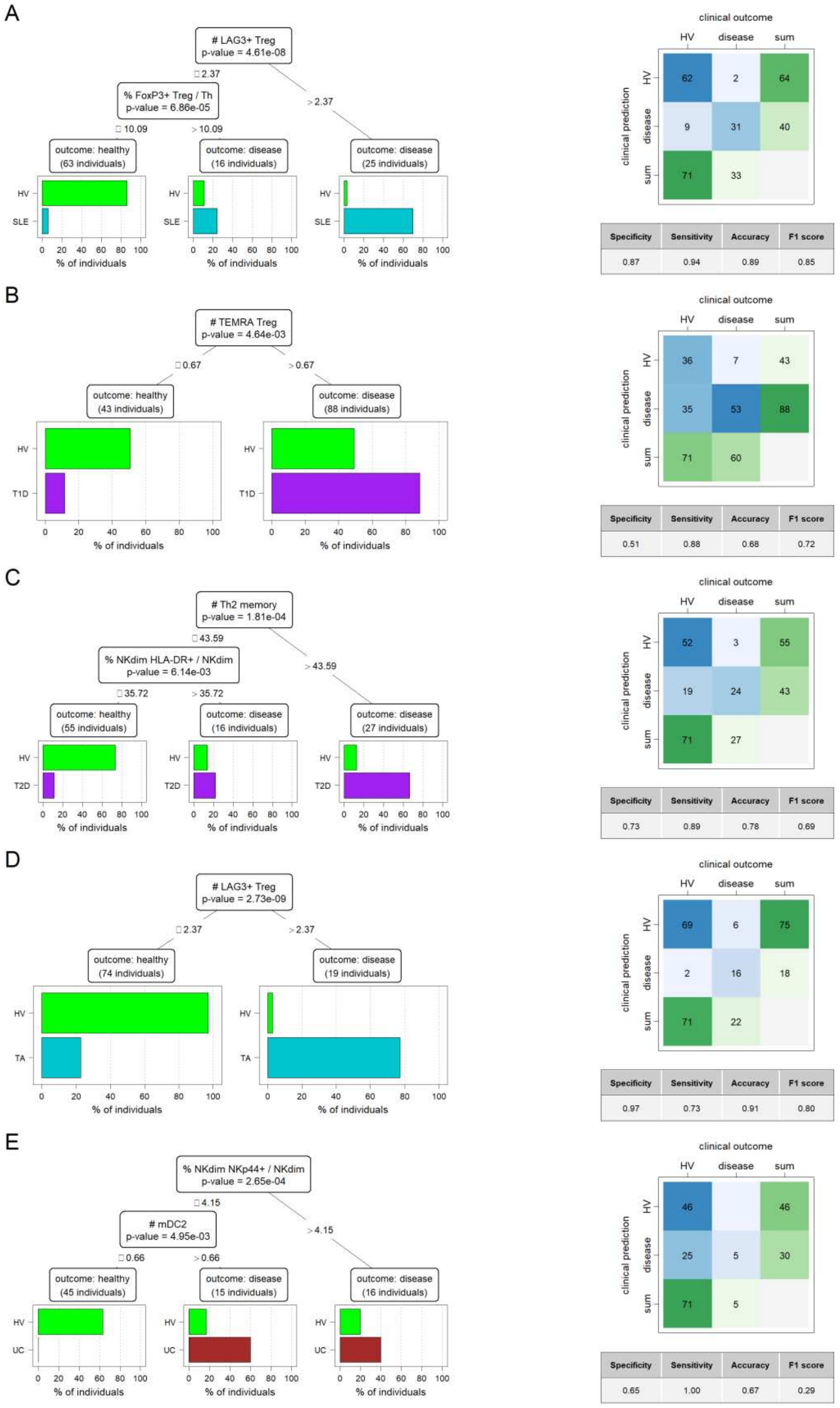

Figure S7. (**A-E**) Classification decision trees constructed to discriminate SLE, T1D, T2D, TA, and UC patients from healthy volunteers. For each classification leaf, the percentages of well-classified patients and well-classified individuals are indicated. The specificity, sensitivity, accuracy, and F1 score measures associated with each model are indicated along with the confusion matrix.

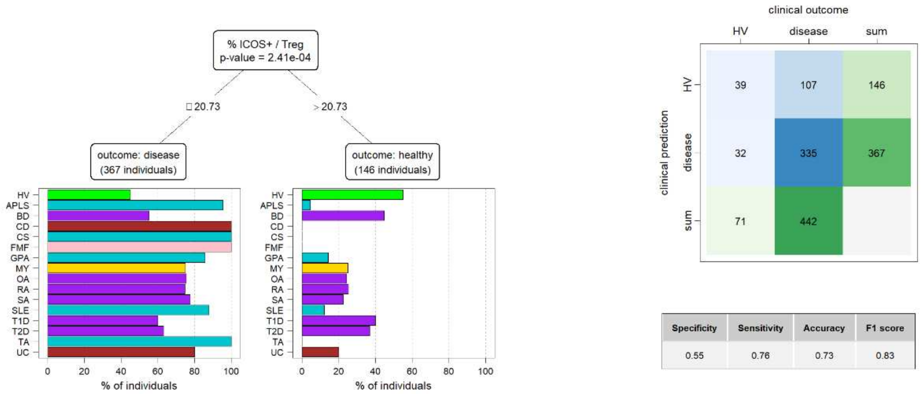

Figure S8. Classification decision tree constructed to discriminate all patients – regardless of disease – from healthy volunteers. For each classification leaf, the percentages of well-classified patients and well-classified individuals are indicated. The specificity, sensitivity, accuracy, and F1 score measures associated with the model are indicated along with the confusion matrix.

Supplementary references

1 Miyakis S, Lockshin MD, Atsumi T, *et al.* International consensus statement on an update of the classification criteria for definite antiphospholipid syndrome (APS). *J Thromb Haemost*. 2006;4:295–306.

2 Criteria for diagnosis of Behçet’s disease. International Study Group for Behçet’s Disease. *Lancet*. 1990;335:1078–80.

3 International Team for the Revision of the International Criteria for Behçet’s Disease (ITR-ICBD). The International Criteria for Behçet’s Disease (ICBD): a collaborative study of 27 countries on the sensitivity and specificity of the new criteria. *J Eur Acad Dermatol Venereol*. 2014;28:338–47.

4 Masi AT, Hunder GG, Lie JT, *et al.* The American College of Rheumatology 1990 criteria for the classification of Churg-Strauss syndrome (allergic granulomatosis and angiitis). *Arthritis Rheum*. 1990;33:1094–100.

5 Livneh A, Langevitz P, Zemer D, *et al.* Criteria for the diagnosis of familial Mediterranean fever. *Arthritis Rheum*. 1997;40:1879–85.

6 Leavitt RY, Fauci AS, Bloch DA, *et al.* The American College of Rheumatology 1990 criteria for the classification of Wegener’s granulomatosis. *Arthritis Rheum*. 1990;33:1101–7.

7 Bohan A, Peter JB. Polymyositis and dermatomyositis (first of two parts). *N Engl J Med*. 1975;292:344–7.

8 Bohan A, Peter JB. Polymyositis and dermatomyositis (second of two parts). *N Engl J Med*. 1975;292:403–7.

9 Kellgren JH, Lawrence JS. Radiological Assessment of Osteo-Arthrosis. *Annals of the Rheumatic Diseases*. 1957;16:494–502.

10 Aletaha D, Neogi T, Silman AJ, *et al.* 2010 Rheumatoid arthritis classification criteria: an American College of Rheumatology/European League Against Rheumatism collaborative initiative. *Arthritis Rheum*. 2010;62:2569–81.

11 Kay J, Upchurch KS. ACR/EULAR 2010 rheumatoid arthritis classification criteria. *Rheumatology*. 2012;51:vi5–9.

12 Rudwaleit M, van der Heijde D, Landewé R, *et al.* The development of Assessment of SpondyloArthritis international Society classification criteria for axial spondyloarthritis (part II): validation and final selection. *Ann Rheum Dis*. 2009;68:777–83.

13 Petri M. Review of classification criteria for systemic lupus erythematosus. *Rheum Dis Clin North Am*. 2005;31:245–54, vi.

14 Arend WP, Michel BA, Bloch DA, *et al.* The American College of Rheumatology 1990 criteria for the classification of Takayasu arteritis. *Arthritis Rheum*. 1990;33:1129–34.

15 American Diabetes Association. Diagnosis and classification of diabetes mellitus. *Diabetes Care*. 2014;37 Suppl 1:S81-90.

16 American Diabetes Association. 2. Classification and Diagnosis of Diabetes: Standards of Medical Care in Diabetes—2021. *Diabetes Care*. 2020;44:S15–33.
